# Fibroblast growth factor receptor 3 effects on proliferation and telomerase activity in sheep growth plate chondrocytes

**DOI:** 10.1186/2049-1891-3-39

**Published:** 2012-12-07

**Authors:** Logan B Smith, Janelle M Belanger, Anita M Oberbauer

**Affiliations:** 1 Department of Animal Science, University of California, Davis, CA, 95616, USA

**Keywords:** Chondrocytes, Growth-plate, Telomerase, Fibroblast growth factor receptor 3, Thyroid hormone, Sheep

## Abstract

**Background:**

Fibroblast growth factor receptor 3 (FGFR3) inhibits growth-plate chondrocyte proliferation and limits bone elongation. Gain-of-function FGFR3 mutations cause dwarfism, reduced telomerase activity and shorter telomeres in growth plate chondroyctes suggesting that FGFR3 reduces proliferative capacity, inhibits telomerase, and enhances senescence. Thyroid hormone (T_3_) plays a role in cellular maturation of growth plate chondrocytes and a known target of T_3_ is FGFR3. The present study addressed whether reduced FGFR3 expression enhanced telomerase activity, mRNA expression of telomerase reverse transcriptase (TERT) and RNA component of telomerase (TR), and chondrocyte proliferation, and whether the stimulation of FGFR3 by T_3_ evoked the opposite response.

**Results:**

Sheep growth-plate proliferative zone chondrocytes were cultured and transfected with siRNA to reduce FGFR3 expression; FGFR3 siRNA reduced chondrocyte FGFR3 mRNA and protein resulting in greater proliferation and increased TERT mRNA expression and telomerase activity (p < 0.05). Chondrocytes treated with T_3_ significantly enhanced FGFR3 mRNA and protein expression and reduced telomerase activity (p < 0.05); TERT and TR were not significantly reduced. The action of T_3_ at the growth plate may be partially mediated through the FGFR3 pathway.

**Conclusions:**

The results suggest that FGFR3 inhibits chondrocyte proliferation by down-regulating TERT expression and reducing telomerase activity indicating an important role for telomerase in sustaining chondrocyte proliferative capacity during bone elongation.

## Background

Linear bone growth is a function of the proliferative capacity of the endochondral growth plate and the size of the hypertrophic cells. Regulators of chondrocyte proliferation greatly influence the rate and extent of long bone growth and the resulting mature skeletal size [1,2]. Fibroblast growth factor receptor 3 (FGFR3) is a critical regulator of growth plate chondrocyte function through its inhibition of proliferation [3,4]. Gain-of-function mutations in FGFR3 cause severe restriction of skeletal growth resulting in dwarfism in both mice and humans [5]. Loss-of-function mutations in sheep FGFR3 cause skeletal overgrowth through excessive proliferation of chondrocytes in the growth plate [6,7].

The inhibitory regulation by FGFR3 and its localized expression within the growth plate proliferative zone are unique among the family of four fibroblast growth factor receptors. The other FGFRs, 1, 2 and 4, promote proliferation and are primarily expressed in the perichondrium [8,9]. Evidence also suggests that FGFR1 may promote differentiation in the hypertrophic growth plate zone following exit from the proliferative zone [8,9].

Many hormones and growth factors beyond FGFR3 play a role in the function of the growth plate. Early findings from children pointed to a clear association between circulating thyroid hormone (T_3_) and skeletal size [10]. Thyroid hormone recruits resting zone growth plate chondrocytes to initiate proliferation but then inhibits further proliferation and induces hypertrophy to accelerate bone aging (reviewed in [11]); it also induces FGFR3 expression [12]. The inhibitory effects of T_3_ are balanced by growth factors that promote proliferation at the growth plate.

Chondrocytes within the growth plate undergo multiple rounds of proliferation to effect bone elongation [13]. Sustained proliferation of cells can lead to chromosomal degradation and DNA damage after consecutive replications unless telomere length is maintained [14]. Telomeres act as protective caps to the chromosomes and their length is maintained by telomerase, an enzyme consisting of a reverse transcriptase catalytic subunit (TERT) and a template RNA subunit (TR) moiety [15,16]. Several studies using human in vitro models have also demonstrated a growth-promoting role of telomerase and TERT that is independent of telomere-length maintenance [17-19], however this remains controversial [20]. Transfection experiments have shown that up-regulation of telomerase activity enhances proliferation and immortalizes cells whereas down-regulation of telomerase eventually leads to a halt in proliferation following critical telomere erosion [14,21,22].

Growth plate chondrocytes exhibit reduced proliferative capacity and cellular senescence as animals advance through puberty [23]. Although the mechanism controlling this gradual cessation of proliferation in growth plate chondrocytes is not well understood, human chondrocyte proliferation rates correlate with telomerase levels and both decline with advancing age [24]. A gain-of-function FGFR3 mutation in humans is correlated with reduced growth plate proliferation, shorter telomeres, reduced telomerase activity, and down-regulated TERT suggesting that FGFR3 may directly inhibit telomerase [24]. To determine if FGFR3 down-regulates telomerase activity, we hypothesized that reducing FGFR3 expression levels through siRNA would enhance chondrocyte proliferation, TERT mRNA expression, and telomerase activity whereas induction of FGFR3 via the addition of T_3_ would have the opposite effect demonstrating coordination between inhibition of proliferation within the growth plate and conversion to the hypertrophic phenotype.

## Methods

### Cell culture

Costochondral growth plates were grossly excised from two commercial-bred male lambs at one week of age. The University of California, Davis Institutional Animal Use and Care Committee approved the experimental protocols for this study. For each trial, all dissected growth plates were digested to release growth plate chondrocytes as previously described [25] and pooled. Freed chondrocytes were layered onto a discontinuous isotonic gradient as described previously with the 1.0578 g/mL density fraction used as the source of the primary proliferative zone cells [26-28] and plated at 200,000 cells/well in 2 mL of Dulbecco’s modified Eagle’s medium (DMEM)/F12 (Gibco BRL, Grand Island, NY, USA) containing 5% fetal bovine serum (FBS) (Gibco), penicillin (100 U/mL) and streptomycin (100 mg/mL) (JRH Biosciences, Lenexa, KS) in 6 well tissue-culture plates. In all cultures chondrocytes were 98% viable, determined by trypan blue exclusion, at time of plating. After 1 day, the medium was replaced and cells were incubated for another day in complete media. Mitotic doubling time of chondrocytes was approximately 1.5 days. Primary growth plate chondrocytes under similar in vitro culture methods maintain their chondrocyte morphology and are resistant to dedifferentiation through 14 passages [25,29]. In the present experiment, the cells were cultured for approximately one week with fewer than five mitotic doublings and the chondrocyte phenotype was confirmed by using ovine specific PCR primers [30]. Isolated cells expressed type II collagen mRNA throughout the culture period. The entire primary chondrocyte isolation procedure and treatments were repeated at a later time in a replicate of the trial. For each trial, the experimental unit was culture well with each treatment replicated in three wells. The data reported represent the results of the treatment replicates for the two trials. Notably, a trial effect was not discernible statistically.

### siRNA transfection

Proliferative zone chondrocytes were transfected 2 days post plating with double stranded RNA (dsRNA) oligos to mediate post-transcriptional degradation of FGFR3 and FGFR2 mRNA. The siRNA oligos (Table 1) were designed from published mRNA sequences (GenBank: AY737276; AJ320477) using the Stealth RNAi designer (Invitrogen, Carlsbad CA). Proliferative zone chondrocytes were lipofectamine transfected as per kit instructions (BLOCK-IT transfection kit; Invitrogen) at a final concentration of 100 nM dsRNA per well for each oligo sequence transfected. Untransfected chondrocyte cultures were also used as a ‘baseline’ control to account for any direct effects of lipofectamine on treated chondrocytes. A GFP reporter plasmid (fluorescein-labeled, Invitrogen, Carlsbad, CA), cultured in parallel to the treated chondrocytes, was used as a transfection efficiency control. After a 24-hour incubation period post-transfection, transfection efficiency was measured by counting the proportion of strongly fluorescing cells containing the GFP reporter plasmid. The proliferative zone chondrocyte transfection efficiency for the GFP reporter plasmid was approximately 68%. A scrambled dsRNA (ScR) was transfected and used as control cultures for determining the effect of the targeted FGFR3 siRNA knockdown. Growth plate proliferative zone chondrocytes also express minimally FGFR2, a receptor known to promote chondrocyte replication [31]. To eliminate confounding effects of FGFR2 on proliferation all experimental chondrocyte cultures regardless of additional treatments, including the ScR control, were subjected to FGFR2 siRNA knockdown (Table 2).

**Table 1:** Oligo sequences and accession numbers

	**Sequence (5’ to 3’)**	**Strand**	**Accession**
**siRNA oligos**			
FGFR3	CAGGUGUCCUUGGAGUCCAGUUCAU	Sense	AY737276
	AUGAACUGGACUCCAAGGACACCUG	Anti-sense	
FGFR2	GGGAAUAUACGUGCUUGGCGGGUAA	Sense	AJ320477
	UUACCCGCCAAGCACGUAUAUUCCC	Anti-sense	
Scrambled control (ScR)	CAGUGCCUUGGGACUACUGUUGCAU	Sense	Invitrogen
	AUGCAACAGUAGUCCCAAGGCACUG	Anti-sense	
**Real-Time PCR primers**			
FGFR3	CGCAGGACACCAGGTCTTTG	Forward	NM 174318.3
	cggcTACTCCTTCGACACCTGCG	Reverse	
TERT	CGCTCCTTCCTGCTCTGCTC	Forward	NM 001046242.1
	cggacGATGGTTTCCACGAGTGTC	Reverse	
TR	GCAGACTGGATGGTGGATGG	Forward	NR 001576.1
	cggtaCGCTGTGCTTTTGGTTACG	Reverse	
Kanamycin resistance - Exogenous Standard	AAGCCCACTGCAAGCTACCTG	Forward	Invitrogen PCRIII vector based amplicon
	CGTTTTGGCTATCTGGACAAGGGAAA	Reverse	

**Table 2:** Treatments applied to isolated growth plate proliferative chondrocytes: double stranded RNA (dsRNA) oligos to mediate post-transcriptional degradation of FGFR3 (siRNA FGFR3) and FGFR2 mRNA (siRNA FGFR2), scrambled dsRNA as control, 30 pM recombinant human FGF18, or 1 μM tri-iodothyronine (T3)

**Treatment**	**ScR**	**siRNA**	**T3**
**siRNA FGFR3**	-	+	-
**scrambled dsRNA**	+	-	-
**thyroid hormone**	-	-	+
**siRNA FGFR2**	+	+	+
**FGF18**	+	+	+

### Hormonal treatments

Hormonal treatments to enhance FGFR3 expression and activation were given 1 day post siRNA transfection: the lipofectamine-dsRNA transfection solution was removed and replaced with 2 mL/well of complete media containing the appropriate hormonal treatment. Recombinant human FGF18 (30 pM, Peprotech, Rocky Hill NJ), an FGFR3 ligand, was added to all cultures to activate the receptor [32]. To enhance FGFR3 expression 1 μM tri-iodothyronine (T_3_) (SIGMA, St. Louis, MO) was added to mediate transcriptional up regulation of FGFR3 mRNA [12]. Chondrocytes from culture wells were harvested at 3, 5, and 7 days post siRNA transfection by digestion with 2.5% trypsin (without phenol red, calcium, or magnesium) (1 ml/well) for 15 min at 5% CO_2_ and 37°C followed by 3 mg/mL collagenase type II in DMEM/F12/5% FBS (2 mL/well) for 45 min. Following digestion, cell aggregates were gently triturated with a glass pipette, scraped, and collected. The cells were centrifuged at 150 × g to pellet for supernatant removal. Cells were washed in 1X PBS and centrifuged again and the cell pellet snap frozen in liquid nitrogen and stored at −80°C until DNA, RNA, or protein isolation.

### Isolation and quantification of DNA, RNA, and protein

Thawed cell pellets were mechanically lysed in 50 μl 1X PBS by trituration through a small bore pipet tip. The lysate was homogenized using a Qiashredder spin-column according to manufacturer recommendations (Qiagen, Germantown, MD). Approximately 10% of the cell lysate volume was removed, diluted 1:10 in CHAPS lysis buffer, snap frozen in liquid nitrogen, and stored at −80°C. DNA, RNA, and protein were isolated from the remaining aliquot of the cell lysate (All Prep™ kit, Qiagen, Germantown, MD). A Qubit fluorometer based Quant-it assay (Invitrogen, Molecular Probes Inc., Eugene, OR) was used to determine the DNA, RNA, and protein concentration of each well sample; all wells were replicated in triplicate under each treatment. Chondrocyte proliferation was determined by quantifying DNA concentration of chondrocyte cultures at 3, 5, and 7 days post siRNA transfection [33].

### Two-step real-time quantitative PCR (qPCR)

Culture mRNAs were transcribed to cDNA with the iScript cDNA synthesis kit (Bio-Rad, Hercules CA) containing oligo d(t) and random-hexamer primers in a 20 μl reaction (4 μl 5X iScript reaction mix, 1 μl iScript reverse transcriptase, remaining 15 μl water and 500 ng total RNA) as per the manufacturer’s recommended protocol. The resulting reaction volume was diluted 1:5 with DEPC water and stored at −20°C.

Fluorescent-reporter specific primers for real-time qPCR were designed using the Invitrogen online D-Lux designer (Invitrogen, Carlsbad CA) and published mRNA sequences for FGFR3, TR and TERT (Table 1, GenBank: NM_174318.3, NR_001576.1, and NM_001046242.1, respectively). All real-time qPCR reactions were run in 96-well plates using UDG-Supermix (Invitrogen, Carlsbad CA). Each reaction contained 10 μM of each primer (forward and reverse), and 5 μl diluted cDNA, in a final volume of 50 μl. Samples were amplified in an MJ Research Chromo 4 Detector (BioRad, Hercules CA) with one cycle of 50°C for 2 min and 95°C for 2 min, followed by 49 cycles of 95°C for 15 sec, 60°C for 30 sec with a ramp rate of 2°C per sec. Fluorescence was collected during each plate read immediately following the annealing period at 60°C.

The C(t) values were quantified in femtograms using a standard curve equation defined by five serially diluted concentrations of an exogenous cDNA [19]. In this case, the exogenous cDNA standard encoded a kanamycin resistance gene [34] originally constructed as a 1000 bp RNA portion of an Invitrogen pCRII vector. The kanamycin resistance standard RNA was reverse transcribed into cDNA in tandem with the chondrocyte RNA samples it quantified. The C(t) threshold and baseline definitions were held constant for all replicates of a gene signal within each experiment.

### Telomerase activity detection

A TRAPeze Telomerase Detection Kit (Chemicon (Millipore), Temecula, CA) was used to determine telomerase activity in the thawed chondrocyte lysate samples, using real-time quantitative PCR-based amplification of telomeric repeats in an MJ Research Chromo 4 Detector (BioRad, Hercules CA) following the manufacturer’s recommended program. The C(t) values were determined manually by defining the beginning of the linear phase in the log-based fluorescence and the best fit of the standard curve. The C(t) values were quantified in zmoles using a standard curve equation defined by five serially diluted concentrations of a telomerase substrate included in the kit as a quantitation control.

### Western blotting

Equal concentrations of cellular protein isolated as detailed above were suspended in a Laemmli-related sample buffer (ALO, All Prep™ kit, Qiagen, Germantown, MD), incubated at 90°C for 4 min, separated on a 10% SDS-PAGE gel, and transferred to a PVDF membrane (Amersham, GE Healthcare, Piscataway, NJ) by electroblotting (30 V, 0.12 mA/cm^2^ overnight). Following transfer, the PVDF membrane was blocked1 hour in 5% non-fat dry milk/0.1% Tween in TBS, washed for 10 min in 0.1% Tween-20 in TBS, and incubated with 5% BSA and 0.1% Tween-20 in TBS containing either the FGFR3 (0.4 μg/mL, Santa Cruz Biotechnology, Inc., Santa Cruz, CA, #sc-31162) or GAPDH (0.1 μg/mL, Millipore, Billerica, MA #MAB374) primary antibodies for 2.5 hours at 4°C. After primary antibody hybridization, the membranes were washed 3 times with 0.1% Tween-20 in TBS and incubated with a secondary antibody. Immunoblotting was done with horseradish peroxidase-conjugated anti-rabbit or anti-mouse secondary antibodies (Jackson ImmunoResearch, West Grove, PA, 0.04 μg/mL, #711-035-152, #715-035-151) and ECL detection reagents and film according to manufacturer's instructions (Amersham, GE Healthcare, Piscataway, NJ). Immunoblots were digitally captured (Alpha Innotech® ChemiImager™ model 4400) and protein levels were quantified (Alpha Innotech® Spotdenso software for densitometric scanning of bands); GAPDH intensities were used as a loading control and densitometry values adjusted prior to comparing for treatment effects.

### Statistical analyses

For each trial, each treatment was performed in triplicate. The entire trial was replicated using a second preparation of primary chondrocytes. Chondrocyte cell culture data were analyzed with day, trial, siRNA transfection, and T_3_ as the main effects using least squares analysis of variance (ANOVA) to determine statistical significance (PROC GLM, Procedure General Linear Model, SAS version 9.1; SAS Institute Inc., Cary, NC). General linear model analysis of DNA concentration, telomerase activity, mRNA and protein expression levels included Tukey pairwise comparison tests of treatment groups. Statistical significance was defined as P < 0.05 and all data are expressed as the mean ± standard error of the mean.

## Results

### FGFR3 siRNA

**Figure 1:**
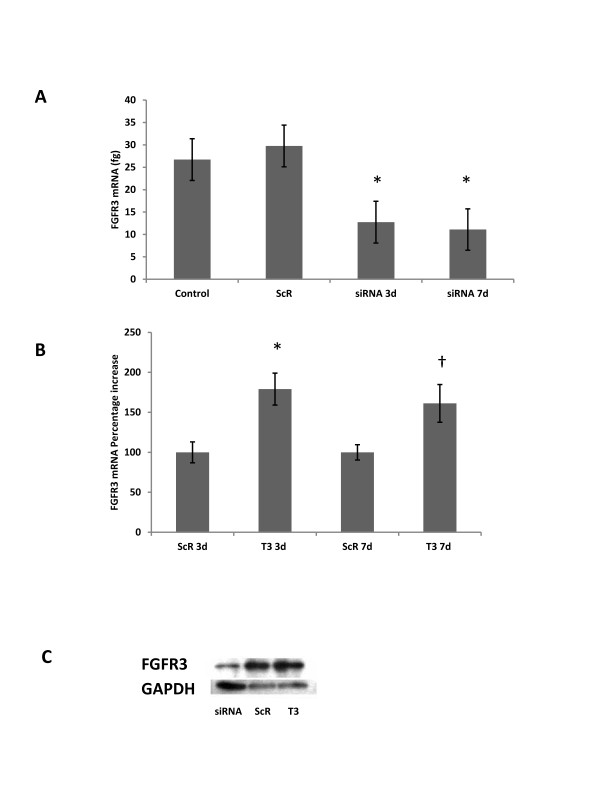
**FGFR3 siRNA and T_**3 **_effects on FGFR3 mRNA and protein.A**) FGFR3 mRNA (femtogram) at 3 (siRNA 3d) and 7 (siRNA 7d) days post siRNA transfection; **B**) FGFR3 mRNA as a percentage of untreated control cells in response to T_3_ treatment at 3 and 7 days post exposure. Data are presented as mean ± SEM. An asterisk (*) denotes means differed from control at p < 0.05; † denotes means differed from control at p < 0.1. **C**) FGFR3 protein levels at 3 days post siRNA transfection for FGFR3 siRNA (lane 1), ScR control treated chondrocytes (lane 2), and T_3_ treated chondrocytes. The blot image is representative of the replicate blots performed and greater amounts of protein were loaded for the FGFR3 siRNA lane to ensure detectable signal.

Untransfected baseline control chondrocytes were not significantly different from ScR control treated chondrocytes, or chondrocytes treated with vehicle controls, for the DNA, mRNA, or protein quantity evaluated at all post transfection days sampled (p > 0.2, data not shown). At 3 days post transfection, cells treated with FGFR3 siRNA had a 57% reduction in FGFR3 mRNA. There was a 55% reduction in FGFR3 protein levels relative to ScR control treated cells (and untransfected controls): 0.61 ± 0.13 and 1.36 ± 0.34 arbitrary densitometric units, respectively (p < 0.05, Figure 1). The reduction in FGFR3 mRNA persisted through 7 days post transfection.

In response to T_3_ treatment, FGFR3 mRNA was increased (p < 0.05, Figure 1). At 3 and 7 days post transfection, T_3_ treated cells had increased FGFR3 mRNA levels relative to cells without T_3_ exposure of 79.1%, and 61.2%, respectively, FGFR3 protein levels followed the increase in mRNA: 3.05 ± 0.84 and 1.36 ± 0.34 arbitrary densitometric units for T_3_ treated cells relative to ScR control treated cells, respectively (p < 0.05, Figure 1).

### Chondrocyte proliferation

To examine the effects of FGFR3 on chondrocyte proliferation in FGFR3 siRNA treated cells, DNA concentration was used as a measurement of cell number. In preliminary experiments we established a direct correlation between cell counts and DNA concentration. Quantifying DNA concentration has also been shown to be a robust indicator of cell number [35]. Seven days post FGFR3 siRNA transfection has been shown to be sufficient time for determining the effects of FGFR3 siRNA on cell proliferation [36]. At 3 and 5 days post siRNA transfection, there was no significant difference in DNA concentration between ScR and FGFR3 siRNA, treated chondrocytes (p > 0.2, Table 3). At day 5, T_3_ treated chondrocytes had reduced DNA concentration. However, at 7 days post siRNA transfection chondrocytes treated with FGFR3 siRNA had significantly greater DNA concentration (p < 0.05), hence cell number, relative to the T_3_ and ScR control treatments with the latter two treatments not differing from one another (p > 0.2).

**Table 3:** Chondrocyte proliferation as indicated by DNA concentration (μg/mL) in response to siRNA transfected, scrambled control (ScR) transfected, or thyroid hormone (T3) treatment over time

	**Treatment**		
	**ScR**	**siRNA**	**T3**
Day 3	13.8 ± 1.9	17.0 ± 1.5	15.7 ± 2.4
Day 5	28.7 ± 0.6	26.3 ± 1.0	15.8 ± 1.0*
Day 7	34.3 ± 1.8	52.9 ± 2.9*	34.2 ± 0.6

* asterisk denotes difference (p < 0.05) between treatment means for a given day; data are presented as mean ± SEM.

### Telomerase expression and activity

**Figure 2:**
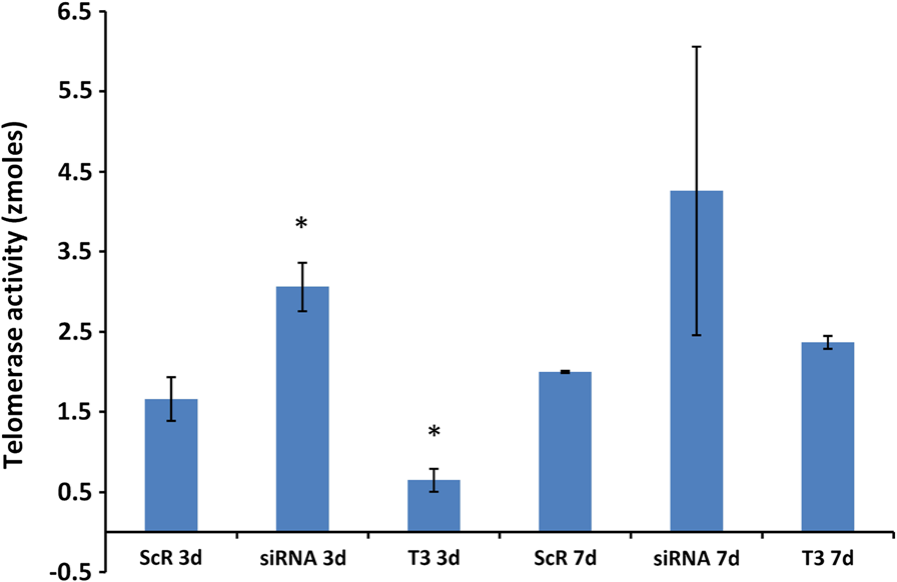
**FGFR3 effects on telomerase activity.** Proliferative zone chondrocytes treated with siRNA to reduce FGFR3 and T_3_ to increase FGFR3 were compared to ScR controls. Data are presented as means ± SEM. An asterisk (*) designates that means for a given day differed from ScR control at p < 0.05. Untransfected chondrocyte cultures were not significantly different from ScR controls (data not shown).

Chondrocytes treated with FGFR3 siRNA had elevated telomerase activity compared to that detected for control chondrocytes whereas chondrocytes treated with T_3_ showed a reduction in telomerase activity relative to the ScR control at 3 days (p < 0.05). Specifically, FGFR3 siRNA chondrocytes had ~80% more telomerase activity than ScR chondrocytes, which in turn had more than 3-fold greater telomerase activity than chondrocytes treated with T_3_ at 3 days post siRNA transfection (p < 0.05, Figure 2). The difference was not maintained at 7 days. Expression of telomerase subcomponent TERT was significantly increased in response to siRNA at 3 days post exposure (1.83 ± 0.27 and 0.85 ± 0.07 femtogram for siRNA and ScR, respectively; p < 0.05). In contrast, exposure to T_3_ did not significantly alter TERT (0.85 ± 0.07 and 0.56 ± 0.24 fg for ScR and T_3_, respectively; p = 0.2). Real-time qPCR analysis of TR mRNA levels showed no difference for the siRNA, ScR control, and T_3_ treatments: 244.57 ± 49.40, 262.90 ± 49.40, 250.20 ± 49.40 femtogram, respectively (p = 0.8), on day 3.

## Discussion

The purpose of the present study was to experimentally evaluate effects of altered FGFR3 expression on proliferation and telomerase activity in sheep growth plate chondrocytes. Wild type growth plate chondrocytes were used in the present study instead of chondrocytes from FGFR3 loss-of-function mutants to more tightly model normal physiological conditions. The knockdown level of approximately 55% achieved in this study has been previously characterized as sufficient for downstream effects of other proliferation inhibitor genes such as p53 [37]. By reducing FGFR3 expression through siRNA transfection, chondrocytes exhibited enhanced proliferation when released from FGFR3 mediated growth inhibition, a finding similar to that seen in human adenocarcinoma cells [36].

To enhance FGFR3 expression, chondrocytes were exposed to T_3_ at doses comparable to published studies [12,38-41]. Chondrocytes treated with T_3_ had double the FGFR3 mRNA levels relative to that detected in controls. Surprisingly, there was no significant sustained effect of T_3_ on cell proliferation in response to the increased FGFR3. Though reduced chondrocyte proliferation was expected upon exposure to T_3_, studies have reported that monolayer chondrocyte cultures respond to T_3_ treatment with increased maturation rather than altered proliferation [41]. The T_3_ effect on chondrocyte maturation is suggested to be mediated by FGFR3 [12]. In humans with genetically over-expressed FGFR3, chondrocyte maturation was correlated with reduced telomerase activity [24]. For the present study, T_3_ was employed to increase FGFR3 expression within proliferative zone chondrocytes and resulted in a down regulation of telomerase activity.

With differentiation of growth plate chondrocytes, telomerase activity would be expected to decline [24]. The target for telomerase down-regulation in mammals is the reverse transcriptase catalytic subunit (TERT) [15,42]. The proliferative chondrocytes of the present study showed ubiquitous TR expression and low levels of TERT mRNA expression in chondrocytes with normal FGFR3 expression. Reducing FGFR3 increased proliferation and telomerase activity suggesting that reducing levels of FGFR3 can increase the activity of telomerase sufficiently to support the replication of the chondrocytes. Ectopic expression of TERT has been shown to enhance proliferation and immortalize cells in culture [17-19]. Elevated TERT expression observed with reduced FGFR3, and lower telomerase levels when chondrocytes had elevated FGFR3 in response to T_3_ suggest that induction of FGFR3 mediates T_3_ action as a negative regulator of telomerase. It must be noted however that other growth factors are influenced by elevated T_3_[10] and the telomerase effects observed may also reflect the contributions of other factors.

Most somatic cells diminish telomerase activity after prenatal development and begin a process of gradual telomere erosion with each replication cycle known as the “mitotic clock” [15,16]. In contrast, tissues requiring rapid and continual proliferation maintain telomerase activity to conserve the integrity of chromosomal structure through sustained replication events [15,16]. Growth plate chondrocytes have active telomerase [43], yet exhibit senescence at an earlier age relative to most somatic cells [23] undergoing only 3 to 5 rounds of mitosis before differentiation and eventual apoptosis [13]. Early senescence and few replicative cycles does not support the need to maintain chromosomal ends and sustained telomerase activity. This would imply that telomere maintenance should not be required in proliferative zone chondrocytes. Yet, telomerase activity does increase with differentiation and progression of chondrocytes through the resting, proliferative, and hypertrophic zones [24]. Taken together, that would suggest an alternate role for telomerase within the growth plate that may involve cellular proliferation [18] as well as promoting differentiation [24,44].

Telomerase has been shown to increase proliferation and delay apoptosis in cells [18], whereas FGFR3 promotes apoptosis and cessation of proliferation in the growth plate [1,45]. In vivo, the presence of a single functional FGFR3 copy results in enhanced skeletal growth and delayed maturation [6,7]. Reduced FGFR3 expression may enhance telomerase activity, increase growth plate chondrocyte proliferation and delay apoptosis, thereby enabling greater endochondral bone growth resulting in a larger mature skeletal size. The opposite would also be true; thus, pharmaceutical interventions used in the down-regulation of telomerase to control cancer [46] may impact growth plate function if given to juveniles. The role of telomerase in both supporting proliferation and then in the subsequent cessation of proliferation are areas that needs further exploration and such studies may shed light on the mechanisms regulating closure of the growth plate.

## Conclusions

The present study demonstrated that reduced FGFR3 expression confers increased proliferative capacity on growth plate chondrocytes through enhanced TERT levels in vitro and suggests a likely translation to enhanced overall bone length in vivo. Collectively the data suggest that normal FGFR3 inhibits cell proliferation by reducing telomerase through down regulating TERT expression and telomerase activity, indicating an important role for telomerase in sustaining chondrocyte proliferative capacity and rate during bone elongation. Further, these findings suggest that the action of T_3_ on growth plate chondrocyte function may be partially mediated by FGFR3 and its effects on telomerase. Although regulation of chondrocyte activity as the growth plate approaches closure is incompletely understood, results of the present study suggest that FGFR3 may play a pivotal role.

## Abbreviations

BSA: Bovine serum albumin; cDNA: Copy DNA; CHAPS: 3[(3-Cholamidopropyl) dimethylammonio]-propanesulfonic acid; DNA: Deoxyribonucleic acid; DEPC: Diethylpyrocarbonate; DMEM: Dulbecco's modified Eagle's medium; ECL: Enhanced chemiluminescence; FBS: Fetal bovine serum; FGF: Fibroblast growth factor; FGFR: Fibroblast growth factor receptor; GAPDH: Glyceraldehyde 3-phosphate dehydrogenase; mRNA: Messenger RNA; PBS: Phosphate buffered saline; PCR: Polymerase chain reaction; PVDF: Polyvinylidene fluoride; qPCR: Quantitative PCR; RNA: Ribonucleic acid; ScR: Scrambled DNA transfection control; siRNA: Small interfering RNA; TBS: Tris buffered saline; T3: Tri-iodothyronine; TERT: Reverse transcriptase catalytic subunit of telomerase; TR: Template RNA subunit of telomerase; Zmoles: Zepto moles (x10^-21^).

## Competing interests

The authors declare that they have no competing interests.

## Authors’ contributions

LBS participated in study design, carried out the experiment, and drafted the manuscript; JMB provided input in study design, analysis, and drafting the manuscript; AMO participated in study design, analysis, study coordination, and drafting the manuscript. All authors have read and approved the final manuscript.
